# PW03-036 – Neutrophilic urticaria with systemic inflammation

**DOI:** 10.1186/1546-0096-11-S1-A262

**Published:** 2013-11-08

**Authors:** H Belani, K Leslie

**Affiliations:** 1 Dermatology, UCSF, San Francisco, USA

## Introduction

Predominantly neutrophilic inflammatory infiltrates are seen in a subset of chronic urticaria patients, with lesions that tend to be less itchy and poorly responsive to antihistamine therapy.[1,2] There have been reports of patients with neutrophilic dermatoses presenting with extracutaneous inflammation.[3] We present two patients with neutrophilic urticaria and extracutaneous symptoms likely mediated by interleukin 1 (IL-1) and not associated with a known connective tissue disease. We propose the term “neutrophilic urticaria with systemic inflammation” (NUSI) to describe a spectrum of diseases, which includes neutrophilic urticaria, and highlights the role of IL-1 in driving this particular inflammatory process.

## Case report

Patient 1: A 47-year old female presented with urticaria and associated night sweats, fevers and polyarticular arthritis. Acute phase reactants were elevated with worsening of symptoms. Initial treatment with topical and systemic corticosteroids, antihistamines, and immunosuppressants was unsuccessful. 100% clinical resolution was achieved with anakinra, an Il-1 receptor antagonist. Patient 2: A 26-year old female presented with urticaria and associated joint pain and swelling. Initial treatment included antihistamines, colchicine, and dapsone. Only colchicine provided moderate benefit, but was stopped due to significant GI-discomfort. Anakinra was initiated; the patient now has 100% control on daily therapy.

## Discussion

The cases described in this report represent a multi-systemic inflammatory entity: neutrophilic urticaria with systemic inflammation (NUSI). In NUSI, there is abrupt onset of neutrophilic urticaria occurring in tandem with inflammatory arthritis and other systemic symptoms, and complete control of symptoms is achieved with IL-1 blockade. NUSI likely lies on a spectrum with clinically similar autoinflammatory diseases, with IL-1 dysregulation and subsequent neutrophil-driven inflammation leading to cutaneous symptoms progressing from varied severity of neutrophilic urticaria to include Sweet’s syndrome to possibly pyoderma gangrenosum. The diagnosis of NUSI is an important one to consider in patients who present with antihistamine-resistant urticaria in combination with systemic inflammatory symptoms, with IL-1 blockade a viable option for therapy.

## Disclosure of interest

H. Belani: None declared, K. Leslie Consultant for: Novartis
